# Autoimmunity in Focal Segmental Glomerulosclerosis: A Long-Standing Yet Elusive Association

**DOI:** 10.3389/fmed.2020.604961

**Published:** 2020-11-20

**Authors:** Manuel Alfredo Podestà, Claudio Ponticelli

**Affiliations:** ^1^Department of Health Sciences, Università Degli Studi di Milano, Milan, Italy; ^2^Retired, Milan, Italy

**Keywords:** FSGS, immunity, permeability factor, podocytopathy, idiopathic nephrotic syndrome

## Abstract

Focal segmental glomerulosclerosis (FSGS) is a histological term that describes a pathologic renal entity affecting both adults and children, with a wide array of possible underlying etiologies. Podocyte damage with scarring, the hallmark of this condition, leads to altered permeability of the glomerular barrier, which may result in massive proteinuria and relentless renal function deterioration. A definite cause of focal segmental glomerulosclerosis can be confirmed in a minority of cases, while most forms have been traditionally labeled as primary or idiopathic. Despite this definition, increasing evidence indicates that primary forms are a heterogenous group rather than a single disease entity: several circulating factors that may affect glomerular permeability have been proposed as potential culprits, and both humoral and cellular immunity have been implicated in the pathogenesis of the disease. Consistently, immunosuppressive drugs are considered as the cornerstone of treatment for primary focal segmental glomerulosclerosis, but response to these agents and long-term outcomes are highly variable. In this review we provide a summary of historical and recent advances on the pathogenesis of primary focal segmental glomerulosclerosis, focusing on implications for its differential diagnosis and treatment.

Focal segmental glomerulosclerosis is a histological term describing the presence of partial tuft sclerosis (“segmental”) in some of the glomeruli (“focal”) from a renal biopsy specimen. As such, FSGS does not identify a specific disease, but rather a lesion with a wide array of possible underlying etiologies that may lead to protean clinical manifestations. FSGS may affect both children and adults, and currently represents one of the most frequent pathologic entities associated with nephrotic syndrome (1). The pathogenic mechanisms leading to FSGS share a common cellular target, the podocyte, a terminally differentiated cell whose foot processes act as structural parts of the glomerular filtration barrier. Podocyte damage may result from systemic diseases, drug exposure, infections or mutations of genes encoding structural podocyte proteins. Nevertheless, a definite etiology cannot be identified in up to 80% of FSGS cases (2), which historically fall under the classification of “idiopathic” or “primary.” Despite this unifying definition, increasing evidence indicates that primary forms may be caused by several distinct pathogenic processes and could therefore benefit from a targeted treatment. Autoimmunity has been consistently reported as a pivotal player in the pathogenesis of these forms, and recent studies suggest that both humoral and cellular immunity may be involved. In this review, we focus on the immune and molecular aspects of podocyte damage associated with a FSGS pattern of injury and discuss current and novel therapeutic options for patients presenting with this condition.

## Pathogenesis

FSGS is now considered as part of the podocytopathy spectrum of diseases, a term that includes all entities in which the podocyte is the primary target of the underlying pathogenic process (3). Podocytes are terminally differentiated epithelial cells that possess foot processes with a highly organized actin cytoskeleton. Interdigitation of podocyte foot processes is fundamental for the integrity of glomerular architecture and concurs in maintaining glomerular permselectivity to macromolecules. The first manifestation of podocyte injury consists in actin cytoskeleton disorganization, increased podocyte motility and foot process effacement (4), which may be followed by podocyte hypertrophy, detachment, and loss. As podocyte regeneration is limited, this process is often insufficient to compensate large podocyte losses, and frequently results in scar formation (5, 6). Such changes lead to a severe alteration in the glomerular structure, loss of filtration barrier selectivity and variable degrees of proteinuria.

Several factors may concur in causing podocyte damage and ultimately lead to FSGS, many of which have been extensively described. Primary FSGS still remains a diagnosis of exclusion and requires ruling out definite etiologies. Accordingly, the pathogenesis of primary FSGS remains poorly defined. The search for a unifying pathogenic mechanism for these forms has been largely unsuccessful, and it is now evident that primary FSGS entails many different diseases with a common phenotype.

### Maladaptive, Genetic, Infectious and Toxic Risk Factors

Secondary causes of FSGS (Table 1) include all those conditions that result in a low nephron number and/or single-nephron hyperfiltration, which are generally categorized as “maladaptive” FSGS (7). In these forms, glomeruli are submitted to an increased mechanical stress that eventually results in hemodynamic alterations, dysfunctional reparative processes and focal sclerosis (8).

**Table 1:** Secondary causes of FSGS.

Genetic	Mutations in genes coding for podocyte proteins Mutations in syndromal genes (including collagen) Risk allele variants (APOL1)
Infections	Human Immunodeficiency Virus Cytomegalovirus SARS-CoV-2 Parvovirus B19 Epstein-Barr Virus Simian virus 40
Drugs and toxins	Heroin and Cocaine Anabolic Steroids Interferon Lithium Pamidronate Sirolimus Calcineurin Inhibitors
Maladaptive—reduced nephron number	Reflux nephropathy Surgical renal ablation Renal dysplasia Unilateral renal agenesis Oligomeganephronia
Maladaptive—normal nephron number	Obesity Hypertension Sickle-cell disease Atheroembolic disease Primary/secondary glomerular disease

A constantly increasing number of mutations in genes encoding for podocyte proteins has been described in both *de novo* and hereditary forms of FSGS (3). In addition, susceptibility genes such as the APOL1 variant are important risk factors for FSGS in selected populations (9, 10).

Interferon (11) and bisphosphonates (12, 13) have been shown to induce severe forms of FSGS, which may sometimes respond to drug cessation and glucocorticoids. Cases of FSGS associated with severe tubulointerstitial lesions were also reported in patients taking cocaine, heroin, calcineurin inhibitors, or lithium (14–16). Podocytopathies with an FSGS pattern can be also caused by HIV, SARS-CoV-2, Parvovirus B19, cytomegalovirus and Epstein–Barr virus (17–20).

### Circulating Permeability Factors

**Figure 1:**
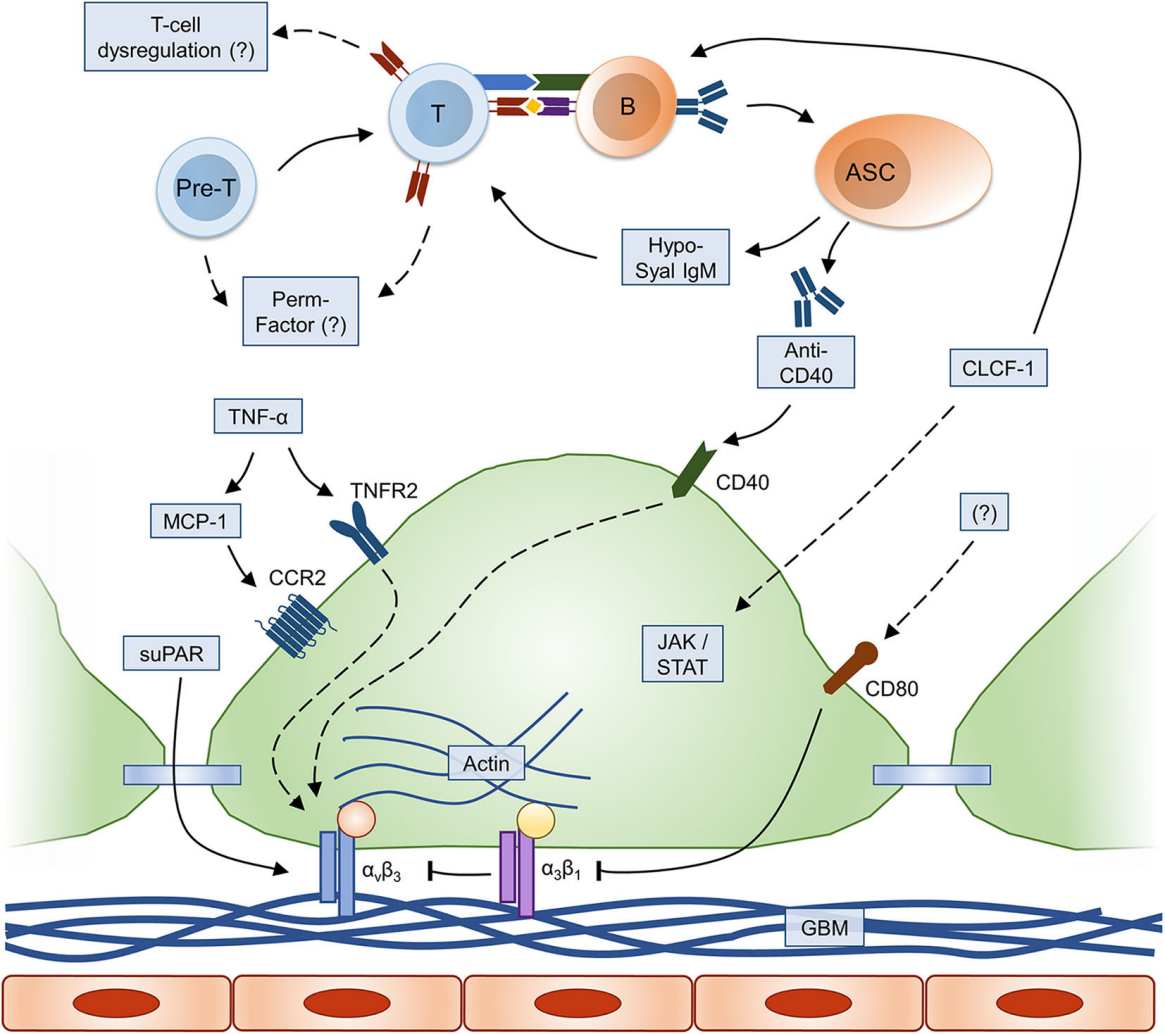
Immune and molecular mechanisms of FSGS pathogenesis. Relevant immune and inflammatory pathways leading to alterations in podocyte foot process architecture are summarized (dashed lines: hypothetical/incompletely understood pathway); please refer to text for explanation. ASC, antibody-secreting cell; B, B cell; CCR2, C-C chemokine receptor type 2; CLCF-1, cardiotrophin-like cytokine factor-1; GBM, glomerular basement membrane; JAK/STAT, Janus kinases (JAK) and signal transducer and activator of transcription proteins (STAT) signaling pathway; MCP-1, monocyte chemoattractant protein-1; Pre-T, T-cell precursor; suPAR, soluble urokinase plasminogen activator receptor; T, T cell; TNF, tumor necrosis factor; TNFR2, TNF receptor 2.

Several lines of evidence indicate that one or more molecules that directly or indirectly alter glomerular permeability may be responsible for FSGS in most primary forms (Figure 1). The existence of such “permeability factors” has been supported by rapid FSGS recurrence within hours from renal transplantation (21), by the efficacy of plasma exchange and selective apheresis methods in treating this condition (22–24), and by disease resolution after graft re-transplantation from a patient with FSGS recurrence to a diabetic recipient (25). Consistently, exposure to serum from patients with post-transplant recurrence was shown to increase glomerular permeability both *in vitro* and in animal models (26, 27). Another indirect evidence came from the observation of transient proteinuria in a child from a mother with FSGS, which suggested transplacental transmission of a permeability factor that was eventually cleared by the newborn (28).

The existence of a permeability factor in idiopathic nephrotic syndrome was first hypothesized in the early 1970's with reference to minimal change disease (MCD), another glomerular disorder that falls under the podocytopathy classification. At that time, observations such as the absence of immunocomplex deposition, disease sensitivity to steroids and cyclophosphamide, as well as spontaneous remission following measles infection (which suppresses cellular immunity), led to the hypothesis of a pivotal role for T cells in the disease pathogenesis (29). Subsequent studies showed that a glomerular permeability factor was secreted by human T cells from patients with MCD, but this factor could not be conclusively identified (30–32).

Later on, proteomic analysis of sequentially fractionated plasma from patients with FSGS recurrence after renal transplantation led to the identification of cardiotrophin-like cytokine factor-1 (CLCF-1) as a plausible permeability factor candidate (33–35). CLCF-1 is a 22 kDa B-cell stimulating cytokine from the IL-6 family, expressed in secondary lymphoid organs, bone marrow and lymphocytes. This molecule binds with high affinity to protein A and is present at concentrations 100 times higher in plasma from FSGS patients compared to plasma from healthy controls. CLCF-1 has been shown to increase glomerular permeability to albumin *in vitro*, and to reduce nephrin expression in cultured podocytes. Notably, these effects are blocked by CLCF-1-specific antibodies, which supports a direct pathogenic effect; interestingly, CLCF-1 activity is also inhibited by galactose and normal serum (36–38). Despite these promising results, confirmation by other research groups has not been reported to date. External validation of CLCF-1 as a permeability factor in independent patient cohorts is definitely required to clarify the relative impact of this molecule on FSGS pathogenesis and to plan targeted interventions in the future.

A seemingly major breakthrough in FSGS pathogenesis ignited the renal community in 2011, when Wei and colleagues identified the soluble urokinase plasminogen activator receptor (suPAR) as a possible circulating permeability factor; suPAR is the cleaved form of a membrane-bound glycoprotein (uPAR) that interacts with podocyte αvβ3 integrins, membrane proteins that connect actin filaments with the extracellular matrix. The authors showed that suPAR was increased in most patients with FSGS, and that this molecule activated αvβ3 integrins in cultured podocytes (39), which induced actin filament reorganization and proteinuria (40, 41). Consistently, serum samples obtained before transplant recurrence promoted αvβ3 integrin activation, an effect that was reduced by suPAR removal through plasmapheresis (39), and the extent of podocyte effacement correlated with suPAR levels (42). Moreover, uPAR-null mice treated with high doses of recombinant suPAR developed proteinuria and early FSGS lesions (39). Elevated suPAR levels in 84.3 and 55.3% of FSGS patients were confirmed also in the FSGS CT and PodoNet international cohorts, respectively (43).

Initial enthusiasm was however curtailed by reports from other investigators, who failed to replicate these findings in independent patient cohorts. Serum suPAR concentrations were found to inversely correlate with glomerular filtration rate, and after adjustment for this confounder suPAR lost its ability to discriminate between FSGS and other proteinuric nephropathies (44–49). In addition, subsequent attempts to elicit FSGS changes in wild-type mice were unsuccessful, suggesting that the uPAR-null background could be at least in part responsible for the disease phenotype observed (49–51). Since suPAR can be cleaved into several shorter molecules, some authors suggested that a hypoglycosylated fragment not readily detected by standard assays, rather than full-length suPAR, could be responsible for FSGS pathogenesis (52). In a recent study, a novel method able to identify both full-length and suPAR fragments outperformed the conventional ELISA assay in discriminating FSGS cases from other proteinuric nephropathies in a single-center cohort (53), but external validation has not been reported yet.

Notwithstanding the frequent lack of immune deposits in renal biopsies from FSGS patients, several autoantibodies against selected antigen specificities (actin, adenosine triphosphate synthase, aldose reductase, and angiotensin II type 1 receptor) have been described in anecdotal cases (54, 55). Delville and colleagues analyzed pre-transplant sera from patients with and without post-transplant FSGS recurrence using protein array data. A panel of seven autoantibodies was found to predict disease recurrence with 92% accuracy in a larger validation set. In this panel, autoantibodies against CD40, a costimulatory molecule of the TNF receptor superfamily highly expressed by antigen-presenting cells, bore the strongest impact on the prediction of FSGS recurrence (56). CD40 was found to be expressed by cultured podocytes *in vitro* and in renal biopsies from patients with recurrent FSGS, but not in normal kidneys. Anti-CD40 antibodies isolated from these patients disrupted podocyte architecture *in vitro* and induced proteinuria in wild-type mice, effects that were reversed by a CD40 blocking antibody. Interestingly, blocking either suPAR or αvβ3 integrin activation ameliorated podocyte injury *in vitro* (56), whereas co-administration of suPAR enhanced proteinuria *in vivo* (51, 56, 57), thus suggesting that anti-CD40 antibodies and suPAR may synergize in inducing αvβ3 integrin activation and FSGS lesions. Studies to assess the pathogenicity of the other autoantibodies identified by Delville and colleagues and to validate anti-CD40 antibodies as a permeability factor in additional patient cohorts are eagerly awaited.

### Adaptive Immunity

As previously discussed, the involvement of T cells in the pathogenesis of idiopathic nephrotic syndrome was theorized more than 40 years ago. Since then, multiple studies have evaluated T-cell phenotype and function in these patients, which highlighted differences in the relative abundance of T-cell subsets, skewed polarization toward a T_H_2 phenotype, enhanced mobilization of hematopoietic stem cells, along with increased T_H_17 effector and reduced regulatory T cell frequencies (58–64). Notably, most of these studies were conducted in small cohorts of patients and by pooling histologically disparate podocytopathy cases, thus potentially increasing the risk of simultaneously analyzing the immune phenotype of highly diverse conditions. Interestingly, adoptive transfer of hematopoietic stem cells obtained from patients with FSGS to immunodeficient mice induced foot process effacement and proteinuria; however, these effects were not observed after infusion of peripheral blood mononuclear cells from the same donors, suggesting that immature cells rather than differentiated T cells could be involved in the pathogenesis of the disease (65).

The efficacy of B cell-depleting anti-CD20 monoclonal antibodies in maintaining the remission of steroid-sensitive idiopathic nephrotic syndrome underscored the potential role of B cells in the pathogenesis of MCD- and FSGS-associated podocytopathies (66, 67). Even though the CD20 molecule is not expressed by most antibody-secreting cells (ASC), the depletion of mature and memory B-cells has been shown to hamper the generation of new short-lived ASC, thus potentially impacting on autoantibody production (68). Notably, faster memory B-cell reconstitution after treatment was shown to predict nephrotic syndrome relapse (69), and continuous B cell depletion has been proposed as a strategy to maintain disease remission (70). Within the classification limits of a study in a pediatric cohort with unavailable renal pathology assessment, the production of hypo-sialylated IgM antibodies binding to T-cell surface has been reported as a possible mechanism of steroid dependence in idiopathic nephrotic syndrome (71). This work suggests the existence of a pathogenic link between B and T cells in MCD and FSGS, which can be favorably affected by anti-CD20 therapy. Aside from antibody production, B cell-targeted treatment might also affect the autoreactive T-cell pool, since B cells can efficiently present antigens and provide costimulatory signals to T cells. Moreover, proximity due to interaction with antigen-specific B cells has been proposed as a potential mechanism of autoreactive T-cell depletion following anti-CD20 therapy (72).

The CD80 (B7-1) molecule, which is expressed by antigen-presenting cells and provides costimulatory signals to T cells, has been also implicated in the pathogenesis of FSGS. Lipopolysaccharide-mediated induction of CD80 in podocytes caused actin cytoskeleton reorganization *in vitro* and nephrotic range proteinuria *in vivo* (73). These effects were linked to β1 integrin inactivation, which normally anchors podocyte foot processes to the glomerular basement membrane, and were completely restored by CD80 silencing or pharmacologic blockade (74). CD80 staining in native and post-transplantation renal biopsies identified a subset of patients with FSGS in whom this mechanism seems to be relevant (74), even though these findings were not corroborated by other groups (75, 76). Notably, urinary excretion of CD80 assessed in two large patient cohorts correlated with disease activity and was able to discriminate between primary and secondary FSGS forms, although the highest values were observed for MCD cases (77).

It has also been speculated that podocyte immunological functions could play an important role in damage progression. Indeed, podocytes react to injury by changing their phenotype and setting aside their barrier function in favor of an immunological one; these events determine the alteration of the glomerular barrier and lead to proteinuria. However, in some forms of FSGS, adaptive immunity may also stimulate an autoimmune response that becomes itself an additional source of injury and sensitizes podocytes to circulating factors (78).

### Tumor Necrosis Factor Pathway

Elevated serum concentrations of tumor necrosis factor (TNF)-α have been reported in some patients with FSGS (79), and stimulation of the TNFR2 receptor with TNF-α evoked robust downstream signaling in cultured podocytes (80). However, a subsequent study showed that activation of the TNF pathway in cultured podocytes exposed to serum from FSGS patients occurred in 21% of cases, irrespective of circulating TNF-α levels (81). This once again underscored the heterogeneity of FSGS pathogenesis and identified intrarenal activation of the TNF pathway as a potential convergence point of multiple pathogenic mechanisms in this disease. Similarly to what described for suPAR and anti-CD40 antibodies, TNF pathway stimulation in podocytes induces αvβ3 integrin activation and actin filaments reorganization (82). Evidence of early glomerular TNF pathway activation was also obtained with an unbiased approach, i.e., by microarray analysis of glomerular RNA isolated from pre- to post-transplant biopsies of FSGS patients, suggesting that one or more permeability factors may induce FSGS through this mechanism (83). Consistent with these data, pharmacologic inhibition of TNF-α might be beneficial in some but not all of patients with FSGS (84, 85). Unpublished observations from the NEPTUNE network investigators suggest that patients with activation of the TNF pathway might be at higher risk for rapid renal function deterioration (86). Urinary levels of two downstream components of the TNF pathway, MCP-1 (also known as CCL2) and TIMP-1, were found to be associated with TNF activation in the same cohort (86). Elevated urinary MCP-1 concentrations were also confirmed in FSGS patients by other investigators, and correlated with the degree of proteinuria (87). Moreover, glomerular expression of MCP-1 and its receptor CCR2 were increased in patients with FSGS and in mouse models of the disease (88). CCR2 knockout and antagonism with a small molecule inhibitor reduced glomerular injury and proteinuria *in vivo* (89), thus identifying a possible additional therapeutic target.

## Pathology

The pathognomonic features of FSGS initially affect only a few glomeruli and are characterized by tuft sclerosis, which is limited to a portion of the otherwise normal glomerulus. These lesions are initially predominant in juxtamedullary glomeruli, but progressively spread to the outer cortex. Hence, if renal biopsy is performed early in the course of the disease, FSGS diagnosis may be missed, particularly when the number of sampled glomeruli is small and the biopsy specimen contains only superficial cortex tissue.

The Columbia classification proposed to subdivide FSGS lesions in 5 histologic categories include the tip, cellular, perihilar, collapsing, and the not otherwise specified (NOS) variants (90). However, that classification was based exclusively on light microscopy findings. Different types of lesion may coexist in the same biopsy sample and histologic features can also change over time, with all subtypes usually evolving to a NOS phenotype as renal function deteriorates toward end-stage renal disease (ESRD) (91). The process of segmental sclerosis and capillary collapse progresses to a gradual obliteration of glomeruli, which may undergo complete “reabsorption,” leaving behind non-functioning aglomerular tubules (92). These changes are strongly associated with a progressive form of interstitial fibrosis, tubular atrophy and vascular damage. Some authors also proposed that the collapsing variant may be a completely distinct disease from other FSGS forms, due to its highly unfavorable outcome, peculiar pathology findings and typical association with HIV infection (93).

Immunofluorescence studies are typically negative, but deposits of IgM and C3 may be observed in mesangial and sclerotic areas (9). Strassheim and colleagues hypothesized that natural IgM could bind to neoantigens exposed in the glomerulus due to non-immune injury, activating the complement system and promoting further damage. Consistent with this hypothesis, B cell depletion reduced IgM deposition and attenuated renal injury in a mouse model of FSGS (94). In addition, in a subset of patients with primary FSGS, colocalization of IgM with C3 suggested complement activation following recognition of a cognate antigen (94). However, the clinical significance of these deposits remains controversial (95–97).

On electron microscopy (EM), foot process effacement, podocyte detachment, and segmental sclerosis with podocyte loss are the most common findings. Foot process effacement is often diffuse (>80%) and severe in tip, cellular and collapsing variants, while this feature can be more variable in NOS and less severe in the perihilar variant (2, 98). As discussed later, the degree of foot process effacement has important implications for the differential diagnosis of FSGS causes and for the correct identification of primary forms.

## Clinical Presentation and Outcome

Proteinuria is the most common feature at presentation in FSGS, and may range from sub-nephrotic levels to full-blown nephrotic syndrome with hypoalbuminemia, hypercholesterolemia, and diffuse edema.

Newborns with congenital nephrotic syndrome are usually premature with low birth weight, and severe nephrotic syndrome is diagnosed soon after birth. FSGS is due to genetic mutations in most of these cases, and ESRD typically develops in infancy. These patients have a comparable mortality, growth, and time to transplantation as infants with other primary renal diseases (99).

Children and adolescents frequently present with signs and symptoms of nephrotic syndrome such as periorbital and dependent edema. Owing to the high frequency of steroid-sensitive MCD in this age group, renal biopsy is usually not performed in patients presenting with isolated nephrotic syndrome. Histologic evaluations are usually reserved for those patients with atypical characteristics (syndromic features, rapid renal function deterioration, positive autoimmune panel) and for steroid-resistant cases.

Adults may be asymptomatic, but even sub-nephrotic proteinuria may eventually increase to the nephrotic range over time. Microscopic hematuria is found initially in about half of cases, while gross hematuria is rare. Hypertension is frequent in adults, and impaired renal function may be already present at the time of referral in up to 25% of patients with FSGS. Unless medically contraindicated, renal biopsy should be performed in all adult patients to confirm the diagnosis and to guide future management.

The natural course of primary FSGS unresponsive to treatment is frequently relentless, with 50% of patients progressing to ESRD within 3–8 years from diagnosis (100). In a few cases, FSGS is characterized by a rapidly progressive course marked by massive proteinuria and severe hypertension. Many patients may develop complications due to the nephrotic syndrome, including infection, thrombotic complications, and cardiovascular disease. However, patients who achieve and maintain asymptomatic non-nephrotic proteinuria have a significant improvement in the overall natural history of FSGS, both in term of renal disease progression and extra-renal complications (100, 101).

The best predictor of a favorable outcome is complete remission, which has been defined as proteinuria <0.2–0.3 g/day (or a urinary protein to creatinine ratio <200–300 mg/g) associated with stable glomerular filtration rate (102, 103). Unfortunately, spontaneous complete remission is exceptional, but can be achieved with treatment in a similar proportion of pediatric and adult patients (104). A number of studies pointed out that children (105, 106) and adults (107–109) who achieve complete remission maintain normal renal function over the time, while most of non-responders progress to ESRD. The same investigators outlined that also a partial remission, defined as a proteinuria <2.0–3.5 g/day (variable definition among studies) with stable renal function can improve outcomes in comparison with non-responders. Nevertheless, the length of exposure to proteinuria may be more important than single time-point proteinuria values: time-varying proteinuria has been proposed as a reliable metric to capture the risk of a 50% reduction in glomerular filtration rate or progression to ESRD (110).

Additional clinical factors may provide useful information regarding the prognosis of patients with FSGS. Impaired renal function at presentation indicates a poor prognosis, unless the increase of serum creatinine is the consequence of acute kidney injury, due to diuretic-induced hypovolemia and/or severe hypoalbuminemia. Arterial hypertension can also contribute to the development of renal failure in FSGS: as autoregulation of glomerular pressure in FSGS is impaired, the increase in systemic blood pressure leads to a rise in glomerular pressure, which results in glomerular capillary wall stretch, endothelial damage, and increased filtration of proteins (111) along with microvascular lesions leading to renal ischemia and interstitial fibrosis (112).

Histological findings may also help in assessing renal outcomes. The prognosis is usually severe in patients with diffuse interstitial fibrosis and tubular atrophy (113, 114). In addition, mesangial proliferation at renal biopsy was associated with a 4.6 relative risk of serum creatinine doubling in some series (107). Diffuse and multiple segmental sclerotic areas at the initial biopsy and, even more importantly, an increase in the number of globally sclerotic glomeruli in follow-up biopsies, correlate with chronic kidney disease development.

The Columbia classification may also provide useful prognostic information. The tip lesion variant has been associated with low pathologic scores and rate of progression to ESRD, also due to a high response rate to treatment. Compared to NOS, the collapsing variant usually displays more severe nephrotic syndrome and lower renal function at diagnosis. Overall, 7% of tip, 47% of collapsing and 20% of NOS variant patients progressed to ESRD at 3 years from diagnosis (113). Other studies confirmed that patients with tip lesions display a favorable outcome, while patients with collapsing FSGS have a worse prognosis (115–117).

After renal transplantation, primary FSGS has a high rate of recurrence in the allograft, which significantly reduces long-term graft survival (118). There is considerable variability among case series, but recent data from pediatric and adult cohorts indicate that the overall recurrence rate is similar across age groups, affecting approximately one third of patients (23, 119). These reports may however underestimate the true incidence of primary FSGS recurrence, because most studies defined primary forms irrespective of the presence of nephrotic syndrome or the degree of foot process effacement on EM, likely including secondary FSGS forms in the analysis. Disease remission with treatment can be achieved only in half of cases, which makes recurrent FSGS a largely unmet medical need.

## Diagnosis

A correct differential diagnosis between primary and secondary FSGS forms is paramount to guide management. Even though light microscopy alone cannot differentiate primary from secondary FSGS, primary forms share some typical features, including the presence of a full-blown nephrotic syndrome (proteinuria >3.5 g/day, albuminemia <3.0 g/dL) and EM ultrastructural findings consistent with diffuse (>80%) foot process effacement (Table 2). However, since primary FSGS is still a diagnosis of exclusion, maladaptive, infectious, toxic, and genetic forms should be always ruled out.

**Table 2:** Characteristics of primary and secondary FSGS forms.

	**Primary**	**Genetic**	**Maladaptive**
Clinical presentation	Acute, full-blown nephrotic syndrome in most cases (proteinuria >3.5 g/day, albumin <3.0 g/dL); may develop gradually in some cases	Variable from sub-nephrotic proteinuria to nephrotic syndrome	Gradual development of sub-nephrotic proteinuria (<3.5 g/day), sometimes progressing to nephrotic-range; nephrotic syndrome is extremely uncommon (albumin >3.0 g/dL)
Light microscopy	Can be associated with any variant, glomerulomegaly uncommon	Can be associated with any variant	Often peri-hilar variant, glomerulomegaly is common
Electron microscopy	Diffuse (>80%) foot process effacement	Either diffuse or segmental foot process effacement	Segmental (<80%, often <50%) foot process effacement
Treatment and outcome	Steroids are effective in ~60% of cases, other IS may be used as steroid-sparing agents or for steroid-resistant cases. Lack of response to treatment predicts progression to ESRD	Immunosuppression is typically ineffective, most cases progress to ESRD within a few years from diagnosis	Immunosuppression contraindicated, often good response to RAS-inhibitors; slow progression toward ESRD

*ESRD, end-stage renal disease; IS, immunosuppressive agents; RAS, renin-angiotensin system*.

Hemodynamic maladaptation to a congenital or acquired reduction of nephron mass is responsible for most cases of secondary FSGS. Maladaptive FSGS can be frequently suspected from medical history and renal imaging. Clinically, these forms are characterized by non-nephrotic or nephrotic-range proteinuria in the absence of hypo-albuminemia, hypercholesterolemia, and edema. Maladaptive FSGS frequently shows perihilar hyalinosis involving >50% of hypertrophic glomeruli with segmental lesions. Ultrastructure analysis usually reveals segmental foot process effacement (<80%) instead of the diffuse pattern observed in primary forms, indicating podocyte mechanic injury rather than a circulating pathogenic mediator (98). Infections should be identified by an appropriate work-up and treated accordingly, as remission with disease-specific treatment can be frequently achieved. In drug-induced FSGS, prompt identification and removal of the offending agent are paramount.

The frequency of genetic mutations is high in pediatric patients and tends to reduce with higher age at onset. Genetic testing is usually advised in all patients with congenital nephrotic syndrome and in those presenting with syndromic features and/or a positive family history. Moreover, it was recently proposed that any mismatch between clinical features and ultrastructural findings (i.e., nephrotic syndrome with segmental foot process effacement, or non-nephrotic proteinuria with diffuse foot process effacement) should also trigger genetic testing (7). Nevertheless, the most compelling indication for genetic analyses remains the resistance to an adequately long and correctly dosed steroid course, since a positive result can be obtained in a significant fraction of both pediatric and adult patients in this case (120–122). Further immunosuppressive treatment should be avoided in patients with genetic mutations, as the risk-benefit ratio is unfavorable. In such cases, supportive therapy should be optimized to lessen the impact of additional risk factors (e.g., hypertension) and to manage accompanying symptoms. Despite such measures, most of these patients progress to ESRD over a relatively brief period, but FSGS recurrence after transplantation is virtually non-existent (7), except in cases associated with NPHS1 mutations (123).

## Treatment of Primary FSGS

Asymptomatic patients with non-nephrotic proteinuria and stable renal function usually do not progress to ESRD and are not exposed to the potential complications of nephrotic syndrome. Based on these considerations, no specific treatment besides conservative management (including salt restriction and inhibition of the renin-angiotensin system) is strictly necessary. However, proteinuria, serum creatinine and blood pressure should be monitored over time. Edema, arterial hypertension, dyslipidemia, and hypercoagulability are frequent complications in patients with nephrotic syndrome, whose treatment is critical to improve life-expectancy and quality of life.

The baseline specific treatment for patients with primary FSGS and nephrotic syndrome rests on glucocorticoids. These agents have well-known genomic and non-genomic immunomodulatory properties, which result in a general suppression of cellular and humoral immunity. In addition, glucocorticoids may also directly promote podocyte survival and actin cytoskeleton stabilization, increasing resistance to injury (124, 125). Response to glucocorticoids is crucial to define the prognosis and to guide further management; based on treatment response, patients are classified as steroid-sensitive or steroid resistant (Table 3).

**Table 3:** Clinical definitions in FSGS.

Steroid-sensitive Nephrotic syndrome	Remission of nephrotic syndrome after therapy with glucocorticoids
Frequently-relapsing Nephrotic syndrome	Two or more relapses of nephrotic-range proteinuria within 6 months after initial response to glucocorticoids
Steroid-dependent nephrotic syndrome	Two or more relapses during or within 2 weeks from completion of glucocorticoid therapy
Steroid-resistant nephrotic syndrome	Remission not achieved after adequately dosed glucocorticoid therapy for 4–6 weeks (children) or 16 weeks (adults)

In children, glucocorticoid therapy is started without histologic confirmation and is usually maintained for 2–3 months, with most patients achieving remission within the end of the first month of treatment (126). Of note, the majority of these children have podocytopathies associated with MCD rather that FSGS lesions, which are more likely to respond to steroids. Adult patients may experience a significant delay in the response to glucocorticoids compared to pediatric patients, thus justifying the need of prolonged therapy (prednisone 1 mg/Kg/day or 2 mg/Kg every other day for up to 16 weeks) before being defined as steroid-resistant (SR) (103, 107, 127–129). This label is often mistakenly attributed to patients who are given insufficient doses of glucocorticoids for far too short periods of time. In adult patients who achieve remission (47–66% of cases), glucocorticoids are slowly tapered over the following 6 months (103, 130).

Up to 70–80% of pediatric and adult patients who achieve remission, however, may experience one or more relapses, that are usually treated with the same steroid schedule. These patients may unfortunately become steroid-dependent (SD) or experience frequent relapses (FR) (Table 3), leading to increased exposure to glucocorticoids and their adverse effects. Measures to reduce the risk of steroid toxicity include dose reduction in the elderly and in obese subjects, use of steroid-sparing immunosuppressive agents, and administration of a single dose of a short-acting glucocorticoid in the morning between 7 and 9 a.m., in order to mimic the circadian rhythm of cortisol. Patients should be counseled to maintain regular physical activity to prevent myopathy and obesity, and to follow a low-calorie and low-salt diet to prevent hypertension, edema, obesity and cardiovascular disease (131, 132). *P. Jiroveci* prophylaxis and use of biphosphonates in women over 50 years should be also considered.

Calcineurin inhibitors (CNI) is an additional option for both SR and SD/FR patients, as well as for patients with contraindications to prolonged steroid courses. CNI activity relies on the inhibition of IL-2 signaling essential for T-cell activation; moreover, these agents directly stabilize podocyte synaptopodin, which regulates the actin filament cytoskeleton, and protect against podocyte injury (133, 134). Two small randomized trials (135, 136) and several observational studies (137–140) demonstrated that cyclosporine can significantly reduce proteinuria in SR patients, and similar or even better results have been reported with tacrolimus (141–144). Moreover, cyclosporine efficacy was also demonstrated in SD/FR patients as a steroid-sparing agent (145), even though the relapse rate upon discontinuation can be excessively high. In addition, although the anti-proteinuric effects of CNI are well-demonstrated, there is no established evidence that these agents can prevent FSGS progression in the long-term. Rather, long-term CNI use has been avoided due to fear of development or aggravation of tubular atrophy, interstitial fibrosis, and glomerular sclerosis. This risk may largely depend on the doses used and, although some individuals are particularly prone to CNI toxicity (perhaps because of altered pharmacodynamics), progressive renal damage is less likely to occur in patients with normal kidney function using low CNI doses (<2.5–3.0 mg/Kg/day for cyclosporine and <0.05 mg/Kg/day for tacrolimus).

Cyclophosphamide, an alkylating agent that affects multiple components of the immune system, has showed efficacy when given as treatment of first episodes or in FR patients (146–149), but proved ineffective, at standard doses, in patients with SR nephrotic syndrome (150). Concerns for gonadal and systemic toxicity have discouraged the use of alkylating agents in FSGS, and only a single course is usually recommended (103).

Mycophenolate inhibits *de novo* purine synthesis, preferentially affecting T- and B-cell expansion, but also has non-immune effects that result in prevention of mesangial cell proliferation, inhibition of podocyte apoptosis and preservation of nephrin and podocin expression (151). Mycophenolate showed comparable effectiveness to levamisole in maintaining remission in SD/FR children (152), but was inferior to cyclosporine in another randomized trial (153). Observational studies reported a low rate of remission with mycophenolate in SR patients (154–156). A randomized controlled trial showed that mycophenolate associated with high-dose dexamethasone achieved remission in only one third of patients (157), even though a significant fraction of patients enrolled in the study did not have nephrotic syndrome and were likely affected by secondary FSGS forms. Another trial found that this immunosuppressive agent was inferior to tacrolimus in SR nephrotic syndrome (158). Nevertheless, the use of mycophenolate as maintenance treatment after remission induction with cyclosporine in SR patients might prevent relapses and reduce the risk of nephrotoxicity (159).

Adrenocorticotrophic hormone (ACTH) is a melanocortin peptide that activates melanocortin receptors and controls steroidogenesis, thus inducing immunomodulating and anti-inflammatory effects. Moreover, ACTH has been shown to reduce foot process effacement and podocyte apoptosis, to prevent the downregulation of podocyte-specific proteins and to increase catalase activity, thereby reducing oxidative stress in podocytes (160, 161). Anecdotal reports outlined the possibility of obtaining complete or partial remission with the use of ACTH, either in its synthetic or natural gel form (162–164).

The chimeric monoclonal antibody rituximab and its fully human counterpart ofatumumab selectively deplete B cells through targeting of the CD20 molecule, thus influencing humoral immunity as well as B- and T-cell crosstalk. Moreover, rituximab may also have direct effects on podocytes through SMPDL-3b stabilization, which was shown to prevent the disruption of actin cytoskeleton and podocyte apoptosis (165, 166), although the specificity of this binding has been questioned (167). Rituximab proved to be effective in preventing relapses in both adult and pediatric SD/FR patients (66, 67). On the other hand, poor results from observational studies have been reported with the use of rituximab in SR nephrotic syndrome (168–170), and the only randomized clinical trial available so far failed to detect any additional benefit from rituximab over CNI (171). Ofatumumab efficacy was reported in anecdotal cases of SR nephrotic syndrome (172, 173), but a recent randomized controlled trial in pediatric patients resistant to multiple therapeutic lines was terminated early for futility (174).

Abatacept, a CTLA4-Ig fusion protein with high affinity for CD80, inhibits T-cell costimulation and prevents podocyte β1 integrin inactivation induced by CD80 expression. Abatacept induced partial or complete remission of proteinuria in 4 patients with FSGS recurrence after kidney transplantation and in one patient with FSGS in the native kidney (74). While these data were confirmed by some investigators (175), others were unable to find any beneficial effect with this drug (176, 177). A randomized controlled trial was designed to clarify the effect of abatacept in treatment-resistant nephrotic syndrome (NCT02592798), but was reportedly terminated early due to poor enrolment (85).

Several attempts at non-selective removal or specific blockade of putative circulating factors have been performed, especially in case of post-transplant FSGS recurrence, which represents a particularly challenging and largely unmet medical need. The use of plasma exchange, immunoadsorption and LDL-apheresis methods in patients who did not respond to available therapies have been reported with variable outcomes (178), but methods to selectively remove circulating permeability factor candidates have not been reported yet. A phase 2 randomized clinical trial is under way to assess the effect of the anti-CD40 monoclonal antibody Bleselumab, which blocks the interaction between CD40 and its ligand, in preventing FSGS recurrence (NCT02921789). Based on evidence from *in vitro* experiments of galactose efficacy in antagonizing the effects of CLCF-1, and after preliminary results from anecdotal cases (179), a small randomized controlled trial assessed the effects of oral galactose supplementation in patients with multi-resistant FSGS. Serum galactose concentration did not significantly differ between pre- and post-treatment assessments, as galactose is rapidly metabolized after absorption. A 50% proteinuria reduction with stable renal function was observed in 2 of 7 patients treated, a proportion that was virtually identical to the control arm (180). The same investigators also evaluated TNFα antagonism with adalimumab, reporting an overall response in 4 of 17 patients treated (pooled from the phase 1 pharmacokinetic study and the randomized controlled trial) (84, 180). As activation of the TNF pathway may be a prerogative of only a subgroup of FSGS cases, a trial to assess whether adalimumab can normalize the urinary concentration of TNF pathway activation biomarkers (MCP-1 and TIMP-1) in these patients has been planned (NCT04009668). Moreover, an open label, dose-escalation study to test the efficacy of an oral inhibitor of the MCP-1 receptor CCR2 in adult patients with primary FSGS is currently ongoing (NCT03703908).

## Conclusions

The history of primary FSGS has been characterized by the rise and fall of biomarkers and potential therapeutic targets like very few other disorders in nephrology. Since the term FSGS merely indicates a pathologic entity shared by a wide array of diseases, it is imperative that data from novel therapeutic strategies are obtained from adequately powered trials that appropriately differentiate between primary and secondary FSGS forms (181). In addition, as primary FSGS itself is likely a broad group of disorders with distinctive pathologic mechanisms, efforts should be aimed to the search of novel biomarkers and to the validation of those already proposed in small patient cohorts. In the long run, this may help to define a personalized treatment strategy for each of these patients that could finally surpass the outdated concept of “one-therapy-fits-all.”

## Author Contributions

MP and CP revised the literature, wrote the first draft, and approved the final version of the manuscript. All authors contributed to the article and approved the submitted version.

## Conflict of Interest

The authors declare that the research was conducted in the absence of any commercial or financial relationships that could be construed as a potential conflict of interest.
